# Phenotypic, molecular detection, and Antibiotic Resistance Profile (MDR and XDR) of *Aeromonas hydrophila* isolated from Farmed *Tilapia zillii* and *Mugil cephalus*

**DOI:** 10.1186/s12917-024-03942-y

**Published:** 2024-03-08

**Authors:** Hala F. Ayoub, Ahmed R. khafagy, Aboelkair M. Esawy, Noura Abo El-moaty, Khairiah Mubarak Alwutayd, Abdallah Tageldein Mansour, Reham A. Ibrahim, Dalia A. Abdel-moneam, Reham M. El-Tarabili

**Affiliations:** 1 Department of Fish Health and Management, Central Laboratory for Aquaculture Research (CLAR), Agricultural Research Center, Abo-Hammad, Sharqia, Abbassa 44662 Egypt; 2 Department of Bacteriology, Immunology, and Mycology, Faculty of Veterinary Medicine, Ain Shams University, Cairo, Egypt; 3 Department of Microbiology, Animal Health Research Institute, Mansoura branch, Mansoura, Egypt; 4 Department of Biology, College of Science, Princess Nourah bint Abdulrahman University, P.O. Box 84428, Riyadh, 11671 Saudi Arabia; 5 Fish and Animal Production and Aquaculture Department, College of Agriculture and Food Sciences, King Faisal University, P.O. Box 420, Al-Ahsa, 31982 Saudi Arabia; 6 Fish and Animal Production Department, Faculty of Agriculture (Saba Basha), Alexandria University, Alexandria, 21531 Egypt; 7 Microbiology Department, National Institute of Oceanography and Fisheries (NIOF), Cairo, Egypt; 8 Department of Aquatic Animal Medicine and Management, Faculty of Veterinary Medicine, Cairo University, Giza, 12211 Egypt; 9 Department of Bacteriology, Immunology and Mycology, Faculty of Veterinary Medicine, Suez Canal University, Ismailia, 41522 Egypt

**Keywords:** Prevalence, *Aeromonas hydrophila*, Virulence genes, Antibiotic resistance, cultured freshwater fish

## Abstract

In the present study, *Aeromonas hydrophila* was isolated from *Tilapia zillii* and *Mugil cephalus* samples collected during different seasons from various Suez Canal areas in Egypt. The prevalence of *A. hydrophila*, virulence genes, and antibiotic resistance profile of the isolates to the commonly used antibiotics in aquaculture were investigated to identify multiple drug resistance (MDR) and extensive drug-resistant (XDR) strains. In addition, a pathogenicity test was conducted using *A. hydrophila*, which was isolated and selected based on the prevalence of virulence and resistance genes, and morbidity of natural infected fish. The results revealed that *A. hydrophila* was isolated from 38 of the 120 collected fish samples (31.6%) and confirmed phenotypically and biochemically. Several virulence genes were detected in retrieved *A. hydrophila* isolates, including aerolysin *aer*A (57.9%), *ser* (28.9%), *alt* (26.3%), *ast* (13.1%), *act* (7.9%), *hly*A (7.9%), and *nuc* (18.4%). Detection of antibiotic-resistant genes revealed that all isolates were positive for *bla*_*pse*1_ (100%), *bla*_SHV_ (42.1%), *tet*A (60.5%), and *sul*1 (42.1%). 63.1% of recovered isolates were considered MDR, while 28.9% of recovered isolates were considered XDR. Some isolates harbor both virulence and MDR genes; the highest percentage carried 11, followed by isolates harboring 9 virulence and resistance genes. It could be concluded that the high prevalence of *A. hydrophila* in aquaculture species and their diverse antibiotic resistance and virulence genes suggest the high risk of *Aeromonas* infection and could have important implications for aquaculture and public health.

## Introduction

*Aeromonas* is a ubiquitous Gram-negative bacterial pathogen, considered the causative agent of septicemic diseases, such as hemorrhagic septicemia, epizootic ulcerative syndrome, and motile *Aeromonas* septicemia [1]. However, several species of the genus *Aeromonas* (*Aeromonas sobria, Aeromonas caviae, Aeromonas veronii*) are known to cause fish diseases. *Aeromonas hydrophila* is considered the main pathogen affecting farmed and wild fishes, leading to mass mortalities in aquaculture and mariculture systems with severe losses of millions of dollars annually [2]. *A. hydrophila* has been linked with general clinical signs of septicemia, including extensive hemorrhages, hemorrhages at the base of fins, tail and fin rot, body ulceration, swelling, and abdominal distention [3–5].

The pathogenicity mechanism of *Aeromonas* species is complicated and multifactorial, it is directly correlated to the presence of single or multiple virulence genes that encode extracellular products and toxins, allowing bacterial invasion, multiplication, and colonization in host tissue, thus disease development occurs [6]. So, the molecular detection of these virulence genes is an essential step in determining the potentiality of pathogenic *Aeromonas* [7]. Heat-stable cytotonic enterotoxin (*ast*), heat-labile cytotonic enterotoxin (*alt*), cytotoxic enterotoxins (*act*), aerolysin (*aer*) [7, 8], hemolysin, adhesins, and cytotoxins [9] are the most common virulence genes detected in the pathogenic strains of *A. hydrophila* isolated from different fish species worldwide.

The use of antibiotics in treatment of bacterial diseases is a common practice in the aquaculture sector, it comes as an effort to control such bacterial infections and disease outbreaks in aquaculture. However, the unlimited and widespread inappropriate use of antibiotics in aquaculture for the treatment of bacterial infections result in antibiotic resistance has been developed in numerous fish pathogens globally [10]. Moreover, there is a risk associated with the transmission of the bacteria containing antimicrobial resistance genes from aquaculture to humans via the accumulation of antibiotic-resistant genes in fish by-products [11]. Thus, in the long run, this will inhibit the beneficial microbiota in the human gastrointestinal tract and reduce the effectiveness of antibiotics in treating human diseases [12–15].

The association/combination between virulence factors and antimicrobial resistance genes within *Aeromonas* bacteria is of great concern, as it reflects bacterial fitness and survivability duration and mechanism within its host Ramadan, et al. [16]. Therefore, the aim of this study was to assess the prevalence of potentially pathogenic *A. hydrophila* isolated from *Tilapia zillii* and *Mugil cephalus* collected from the Suez Canal region of the Ismailia governorate, Egypt; through molecular identification of different virulence genes and assess the multiple drug resistance (MDR) and extensively drug-resistant (XDR) genes present in *A. hydrophila* isolates against commonly used antibiotics and antibiotic agents. Then, a pathogenicity test was conducted using *A. hydrophila* isolates selected based on their prevalence of virulence genes, and the survivability and morbidity of infected fish.

## Materials and methods

### Sampling and clinical examination

A total of 120 clinically affected fish (60 *Tilapia zillii* and 60 *Mugil cephalus*) were randomly collected freshly dead or moribund from different private fish farms within Suez Canal areas, Ismailia Governorate, Egypt, during different seasons (*n* = 15 each season/ each species). Fish with external lesions, such as hemorrhages, fin rot, distended abdomen, and skin darkening were transferred in an ice box (-4 ℃) to the laboratory of the Microbiology Department, Animal Health Institute, for immediate bacteriological analysis. General characteristics and clinical signs of all moribund fishes were observed and recorded following Austin, Austin [17].

### Bacterial culturing and isolation

For the detection of *A. hydrophila*, fish external skin surface was first disinfected by spraying with 70% ethyl alcohol before conducting the postmortem examination, as described by Austin, Austin [17]. Kidney, spleen, liver, and gills samples were obtained from each fish and aseptically streaked on Rimler-Shotts (R-S) agar media (HiMedia, India) and *Aeromonas* agar base media (HiMedia, India) supplemented with rehydrated ampicillin (Oxoid^®^, USA), cultured plates were incubation at 29 °C for 18 to 24 h. *A.hydrophila* colonies were picked and subcultured for purification and bacterial morphology assurance analysis according to Quinn, et al. [18]. Then the purified isolates were kept in Tryptic soy broth containing 20% glycerol (v/v) at -20 °C for further biochemical and molecular investigations.

### Bacterial identification

#### Phenotypic characterization

Conventional phenotypic characterization were performed including the following: characterization of colonial morphology (shape and color), Gram staining, motility testing, cytochrome oxidase, catalase, and oxidation fermentation test (O/F). Different isolates were evaluated for sensitivity to novobiocin antibiotic. The hemolytic activity was detected by streaking the bacterial colonies on TSA supplemented with 5% sheep red blood cells Quinn, et al. [18]. The bacterial proteolytic activity was assessed by plating isolates on brain heart infusion agar with 1% egg yolk and incubated at 37 °C for 48 h [19]. The API-20 NE kit (Biomerix, France) is used for further confirmation of retrieved *A. hydrophila* isolates.

### Bacterial genotyping

For molecular identification, genomic DNA was extracted from purified fresh *A.hydrophila* colonies, using the QIAamp^®^ DNA Mini Kit (Cat. No. D4068, Germany) as directed by the manufacturer. The PCR reaction was conducted in a total volume of 25 µl, which comprises 12.5 µl of PCR master mix (Takara, Japan), 1 µl (20 pmol) of each forward and reverse primers (Invitrogen, Carlsbad, CA, USA), 4.5 µl nuclease-free water, and 6 µl (10 ng/µl) of DNA template. The reaction mixture was done in a T3 Thermal cycler, (Biometra GmbH, Göttingen, Germany). *A. hydrophila* strains were primarily confirmed using the *16SrRNA* gene according to Stackebrandt, et al. [20]. A positive control (*A. hydrophila* ATCC 7966) and a negative control (the reaction mixture without a DNA template) were included with each run. A 100 bp (DNA marker) was utilized to determine the appropriate size of the magnified products. The PCR products were electrophoresed on a 1.5% agarose gel containing ethidium bromide (0.5 µg/ml) in Tris borate EDTA buffer and the gel documentation system (Alpha Imager 2200) was used to visualize the gel.

### Sequencing and phylogenetic analysis

The amplified bands of *A. hydrophila* were sequenced, and the sequence was analyzed using the MEGA 11 software program and blasted on NCBI [20]. The sequence obtained from NCBI was imported for multiple sequence alignment using the Clustal W program, followed by phylogenetic tree construction using the neighbour-joining with 1000 bootstrap method following Kumar, et al. [20].

### Antimicrobial susceptibility testing

Twelve antimicrobial agents belonging to seven antimicrobial classes were used to test *A. hydrophila* isolates susceptibility using the disk diffusion method on Muller-Hinton agar (HiMedia, India) according to the Clinical and Laboratory Standards Institute (CLSI) [21]. Antimicrobials tested (Oxoid, Hampshire, England, UK) were ampicillin (AM, 10 µg), amoxicillin-clavulanic acid (AMC, 30 µg) and oxacillin (OX, 5 µg) belonging to β-lactams class. cefadroxil (CFD, 30 µg) and cefotaxime (CTX, 30 µg) belong to class cephalosporines. amikacin (AK, 30 Μg) and gentamicin (GM, 10 µg) belong to the class Aminoglycosides. ciprofloxacin (CIP, 5 Μg) and levofloxacin (LEV, 5 Μg) belong to class fluoroquinolones. Polymyxin B (PB, 10 µg) belongs to the class polymyxins. Doxycycline (DO, 30 µg) belonging to the class tetracyclines and trimethoprim/sulfamethoxazole (SXT, 25 µg) belonging to the sulfonamides class. The test accuracy was determined using *Escherichia coli* ATCC^®^ 25,922 (Manas sas, VA, USA) as a control group. The diameters of the inhibitory zones were evaluated using standards [21]. The multiple antibiotic resistance (MAR) index was evaluated and elucidated for each isolate based on Krumperman [22], using 0.2 as the modal value.

### Virulence and multiple antimicrobial resistance (MAR) genes detection

Molecular identification for detection of virulence was carried out for *A. hydrophila* isolates using specific primers (Invitrogen, Carlsbad, CA, USA) of aerolysin (*aer*A), serine protease (*ser*), *Aeromonas* cytotonic heat-labile enterotoxins (*alt*), *Aeromonas* cytotonic heat-stable enterotoxins (*ast*), cytotoxic enterotoxin (*act*), haemolysin *(hly*A), nuclease (*nuc*) and adhesion (*aha*) virulence genes. The antimicrobial resistance of the retrieved isolated was confirmed by the detection of antimicrobial resistance genes β-lactamase (*bla*_pse1_), β-lactamase (*bla*_SHV_), sulfonamide (*sul1*), tetracycline (*tet*A). The nucleotide sequence and cycling conditions of the used primers are listed in Table 1.

**Table 1:** List of used *A.hydrophila* oligonucleotide primers

Target Gene		Primer sequence (5’-3’)	Amplicon size (bp)	Cycling conditions (35 cycles)			References
				Denaturation	Annealing	extension	
**Virulent genes**							
*Confirmatory gene*	*16SrRNA*	F: AGAGTT TGATCCTGGCTCAG R: GGTTACCTTGTTACGACTT	1200	94 ℃ 1 min	55 ℃ 1 min	72 ℃ 2 min	[63]
*Aerolysin*	*aer*A	F: CACAGCCAATATGTCGGTGAG R: GTCACCTTCTCGCTCAGGC	326	94 ℃ 30 s	52 ℃ 30 s	72 ℃ 30 s	[64]
*Serine protease*	*ser*	F:ACGGATGCGTTCTTTACTCCA R:CCGTTCATCACACCGTTGTAGTCG	211	94 °C for 1 min	64 °C for 30 s	72 °C for 45 s	[65]
*Heat-labile Cytotonic enterotoxin*	*alt*	F: TGACCCAGTCCTGGCACGGC R: GGTGATCGATCACCACCAGC	442	94 ℃ 30 s	55 ℃ 40 s	72 ℃ 45 s	[31]
*Heat-stable Cytotonic enterotoxin*	*ast*	F: TCTCCATGCTTCCCTTCCACT R:GTGTAGGGATTGAAGAGCCG	331				
*Cytotoxic enterotoxin*	*act*	F:AGAAGGTGACCACCACCAAGAACA R:AACTGACATCGGCCTGAACTC	232				
*Haemolysin*	*hly*A	F: GGCCGGTGGCCCGAAGATACGGG R:GGCGGCGCCGGACGAGACGGGG	592	95 °C for 2 min	55 °C for 1 min	72 °C for 1 min	[66]
*Nuclease*	*nuc*	F:CAGGATCTGAACCGCCTCTATCAGG R:GTCCCAAGCTTCGAACAGTTTACGC	504	94 ºC for 1 min	64 ºC for 30 s	72 ºC for 45 s	[65]
*Adhesion*	*aha*	F:GGTATTGTATCCCGGCTCTGTT R:CGGTCCATCGTCGTCCATCTTG	1082	94 ºC for 30 s	60.4 ℃ for 30 s	72 ℃ for 45 s	[67]
**Antimicrobial Resistance genes**							
*β-lactamase*	*bla*_*pse*1_	F: ACC GTATTG AGC CTG ATT R: ATTGAA GCC TGT GTT TGA GCTA	321	96 ℃ 30 s	60 ℃ 30 s	72 ℃ 30 s	[68]
	*bla*_*SHV*_	F: AGGATTGACTGCCTTTTTG R: ATTTGCTGATTTCGCTCG	392	94 ℃ 30 s	54 ℃ 40 s	72 ℃ 40 s	[69]
*Sulfonamide*	*sul1*	F: CGCACCGGAAACATCGCTGCAC R:TGAAGTTCCGCCCAAGGCTCG	163	95 ℃ 15 s	65 ℃ 30 s	72 ℃ 30 s	[70]
*Tetracycline*	*tet*A	F: GCTACATCCTGCTTGCCTT R: CATAGATCGCCGTGAAGAGG	210	95 ℃ 15 s	60 ℃ 30 s	72 ℃ 30 s	[71]

*Initial denaturation of 5 min at 94 ℃. * Final extension at 72 ℃ extended by 10 min

### Pathogenicity test

Two hundred and forty (240) apparently healthy *T. zillii* were obtained from a private fish farm at West Qantara, Suez Governorate, Egypt, with an average body weight of 30.00 ± 3.8 g. The fish were transported to the National Institute of Oceanography and Fisheries, Suez Governorate, Egypt, and acclimated for two weeks in a 1000 L fiberglass tank supplied with de-chlorinated water with continuous oxygen aeration using electric air pumping compressors before the challenge. Fish were fed a commercial pelletized diet twice daily at 3% of their body weight. The water temperature in the aquaria was thermostatically controlled at 26 ± 2 °C [23].After acclimatization, fish were divided into eight groups in duplicate (30 fish/group). The first group received 0.2 mL of sterile normal saline intraperitoneally (IP) as a negative control, whereas the other seven groups received 0.2 mL of an overnight *A. hydrophila* culture at 3 × 10^8^ CFU/mL. The inoculated bacteria was firstly selected for its high virulence and resistance following Kochs postulates, the bacteria was cultivated on tryptic soy broth (Oxoid) at 29 °C for 24 h, then bacterial suspension was prepared and adjusted to the final concentration using a 0.5 McFarland standard and Helber counting chamber. All fish groups were thoroughly inspected daily after the challenge for 2 weeks for any pathological lesions and mortalities [24]. Moribund and freshly dead fish were collected and aseptically examined for bacterial reisolation. At the end of the experiment, the fish were killed by an overdose of anesthesia (200 mg clove oil/L) and hygienically disposed by burning in the incinerator.

### Statistical methods

The distribution data assessments were carried out using the Chi-square test in R-software (version 4.0.2; https://www.r-project.org/), with a significance level of *P* < 0.05.

## Results

### Clinical and postmortem observation

**Fig. 1:**
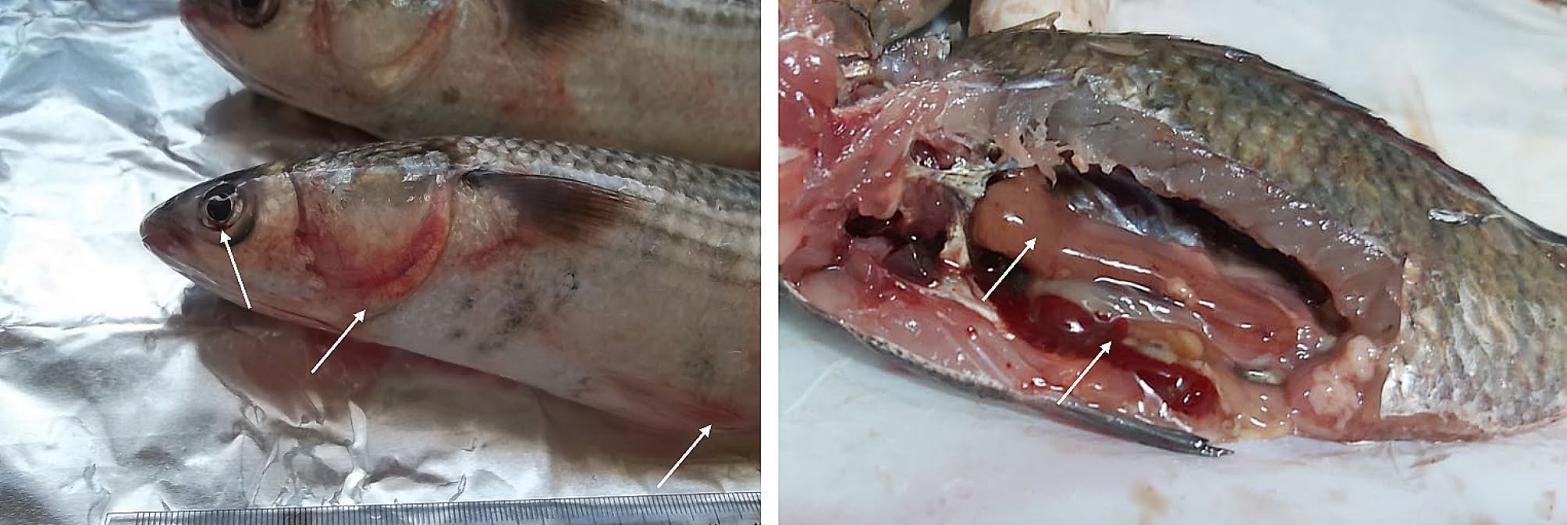
(**A**) Naturally infected *M. Cephalus* showing external hemorrhages on the eye, gill cover and fins, (**B**) Naturally infected *Tilapia zillii* showing congestion, hemorrhages and enlargement of internal organs

Clinical examination revealed that all 120 fish (*T. zillii* and *M. cephalus*) exhibited extensive hemorrhages, hemorrhagic fin erosions, skin darkening, hemorrhages in the eyes and around the gill cover, and some fishes showed abdominal distention. (Fig. 1A). Internally, enlargement and congestion of the internal organs and congested gills were observed (Fig. 1B).

### *Aeromonas hydrophila* isolation and phenotypic characterization

A total of 38 purified *A.hydrophila* isolates were retrieved, on Rimler-Shotts media *Aeromonas* colonies were small, smooth and yellow. On *Aeromonas* agar base media, the colonies were dark green with dark centers. On tryptic soy agar, the colonies were creamy circular, convex, and glistening. On non-lactose fermented MacConkey’s agar, the colonies were pale in color. Moreover, on blood agar, *Aeromonas* colonies showed a beta-hemolytic zone, and are round, grayish and proceeded to dark green color after a long-time incubation. Additionally, all isolates exhibited high resistance patterns against novobiocin.

Microscopically, *A.hydrophila* were gram-negative, short rod-shaped bacilli, and motile with single polar flagella. Conventional biochemical tests revealed that *Aeromonas* is a facultative anaerobic bacteria (O/F +/+) and cytochrome oxidase and catalase tests positive. Moreover, the bacterial Proteolytic activity was evaluated by observing the appearance of a visible proteolytic zone surrounding the bacterial cells cultured on Brain Heart Infusion Agar (HiMedia) with 1% fresh egg yolk and incubated at 30 °C for 48 h.

Based on API-20 NE, the retrieved isolates were confirmed as *A. hydrophila* that react positively to nitrate reduction, glucose assimilation, gelatine liquefaction and negatively to citrate utilization and urease production.

### Bacterial prevalence

*A. hydrophila* was detected in 38 of the 120 examined fish samples with a prevalence percentage (31.6%), 29 isolates were detected in *T. zillii* 48.3% (29/60) and the remaining nine isolates were isolated from *M. cephalus* 15% (9/60). The bacterial isolation from internal organs revealed that *A.hydrophila* were highly prevalent in the liver (16, 42.1%) followed by the kidney (14, 36.8%) and the spleen (5, 13.1%) and prevalence in the gills was the least (3, 7.9%). There was a statistically significant difference in the *A. hydrophila* prevalence among different internal organs of the examined fish (X^2^ = 13.16, *P* < 0.05).

**Fig. 2:**
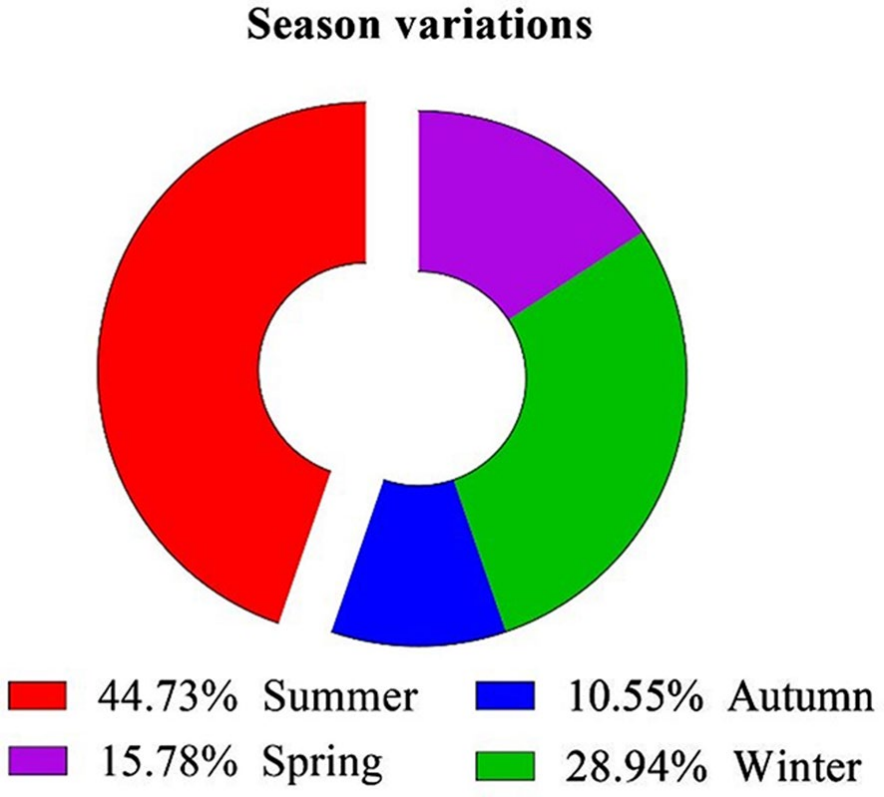
Seasonal variation of *A. hydrophila* prevalence

Seasonally, the prevalence of *A. hydrophila* in naturally infected *T. zillii* and *M. cephalus* varies significantly throughout the year; the summer season recorded the highest percentage of infection (44.73%), followed by winter (28.94%), spring (15.78%) and autumn (10.52%) (Fig. 2). There was a significant difference in *A. hydrophila* prevalence among different seasons (X^2^ = 10.632, *P* < 0.05). Based on the Molecular identification, all the recovered isolates (*n* = 38) were positive for *16SrRNA*.

### Sequencing of the isolated *A. Hydrophila*

**Fig. 3:**
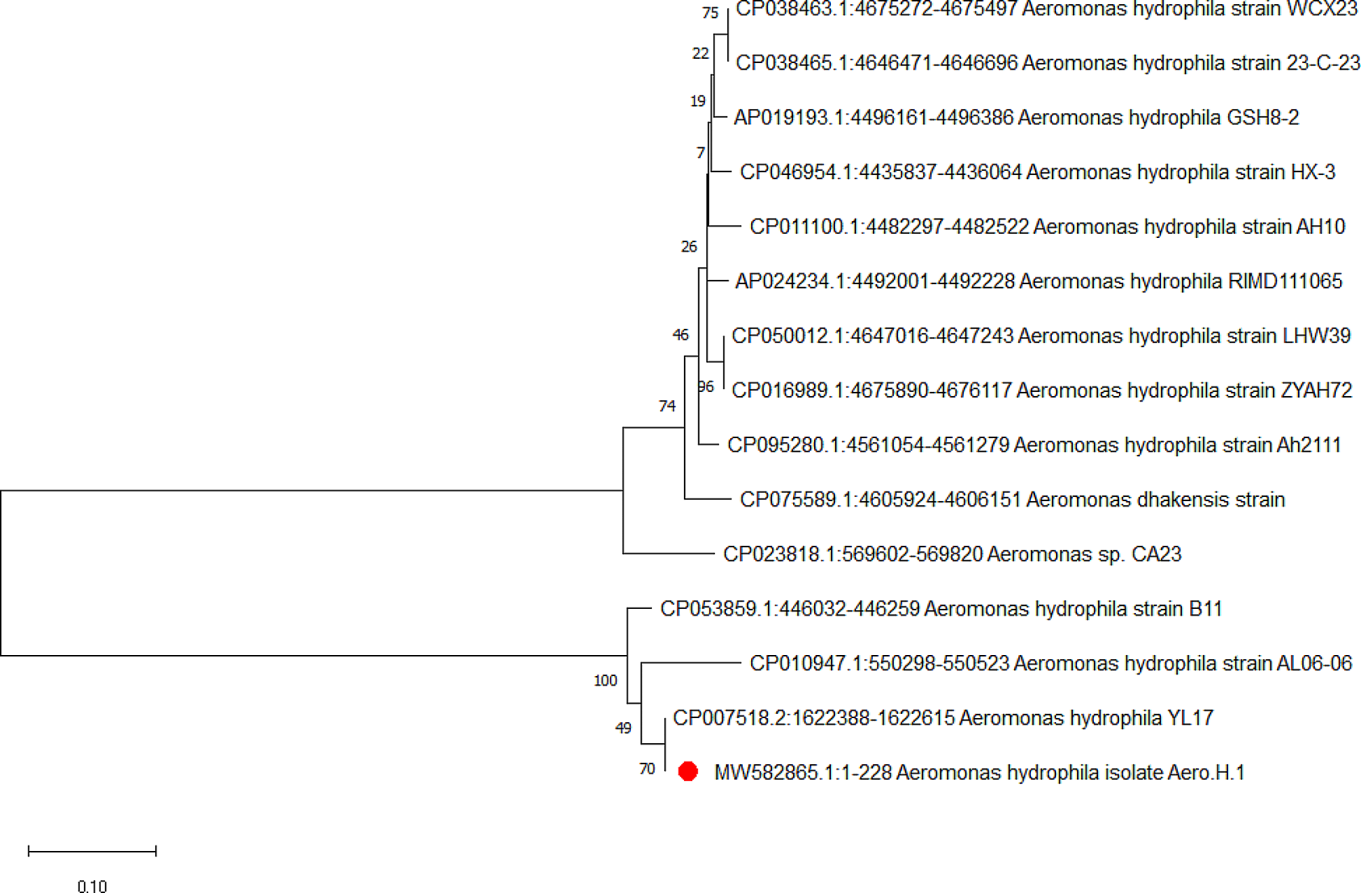
Phylogenetic tree of *Aeromonas hydrophila*

One selected strain for high virulence and resistance was sequenced, and the sequence was submitted to the Gene Bank with accession number (MW582865, https://www.ncbi.nlm.nih.gov/nuccore/MW582865.1/ ). The final alignments showed that isolate MW582865 had a high similarity with strains CP053859, CP028418.1, and CP018201 with a percentage of 96%, 78%, and 77%, respectively. It had a low similarity with strains CP046954, AP024234, CP050012, CP016989.1, and AP019193.1 each with 29%. The derived neighbor-joining phylogenetic tree revealed an apparent clustering of the isolated strain of *A. hydrophila* with various strains of *A. hydrophila* uploaded from the gene bank (Fig. 3). The nucleotide percentage of adenine (A), thymine (T), cytosine (C), and guanine (G) were 17% (39), 16% (33), 34% (79), and 33% (77), respectively (Fig. 3).

### Antimicrobial susceptibility testing

**Fig. 4:**
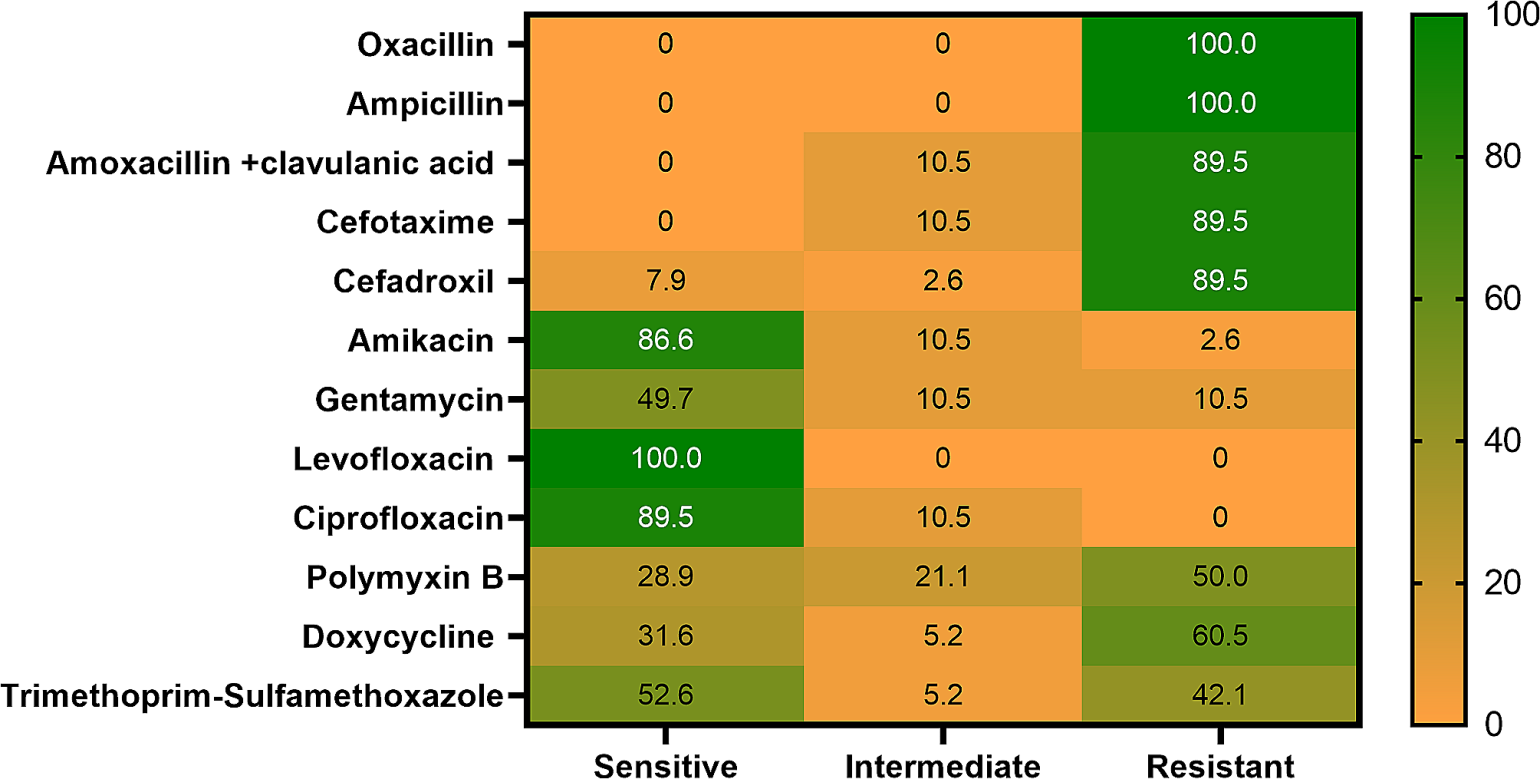
Antibiotic resistance for the recovered *A. hydrophila* isolates

Results of Antibiotic sensitivity of isolated *A.hydrophila* showed that all isolates from *T. zillii* and *M. cephalus* samples displayed a different degree of resistance to all the tested antibiotic agents. The isolates showed exceptional sensitivity to fluoroquinolones; levofloxacin (100%) and ciprofloxacin (89.5%), aminoglycosides; gentamycin (94.7%) and amikacin 86.6% but were highly resistant to *β-lactamase;* oxacillin, ampicillin (100%) and amoxicillin-clavulanic acid (89.5%). cephalosporins;both cefotaxime and cefadroxil (89.5%) (Table 2; Fig. 4). Susceptibility to the different tested antibiotics was statistically significant (*P* < 0.05).

**Table 2:** Antimicrobial susceptibility pattern of *A. hydrophila* isolates (*n* = 38)

Antimicrobial class	Antimicrobial agent	Interpretation					
		Sensitive		Intermediate		Resistance	
		N	%	N	%	N	%
*β-lactamase*	Oxacillin Ampicillin Amoxacillin + clavulanic acid	- - -	- - -	- - 4	- - 10.5	38 38 34	100 100 89.5
Cephalosporins	Cefotaxime Cefadroxil	- 3	- 7.9	4 1	10.5 2.6	34 34	89.5 89.5
Aminoglycosides	Amikacin Gentamycin	33 36	86.6 94.7	4 1	10.5 10.5	1 1	2.6 10.5
Fluoroquinolones	Levofloxacin Ciprofloxacin	38 34	100 89.5	- 4	- 10.5	- -	- -
Polymyxins	Polymyxin B	11	28.9	8	21.1	19	50
Tetracyclines	Doxycycline	12	31.6	2	5.2	23	60.5
Sulfonamides	Trimethoprim-Sulfamethoxazole	20	52.6	2	5.2	16	42.1
Chi-square *P* value		176.14 *P* < 0.0001		25.2 0.008521		138.15 *P* < 0.0001	

### Molecular identification of virulence genes and multiple antibiotic resistance (MAR) genes among *A. hydrophila* isolates

**Fig. 5:**
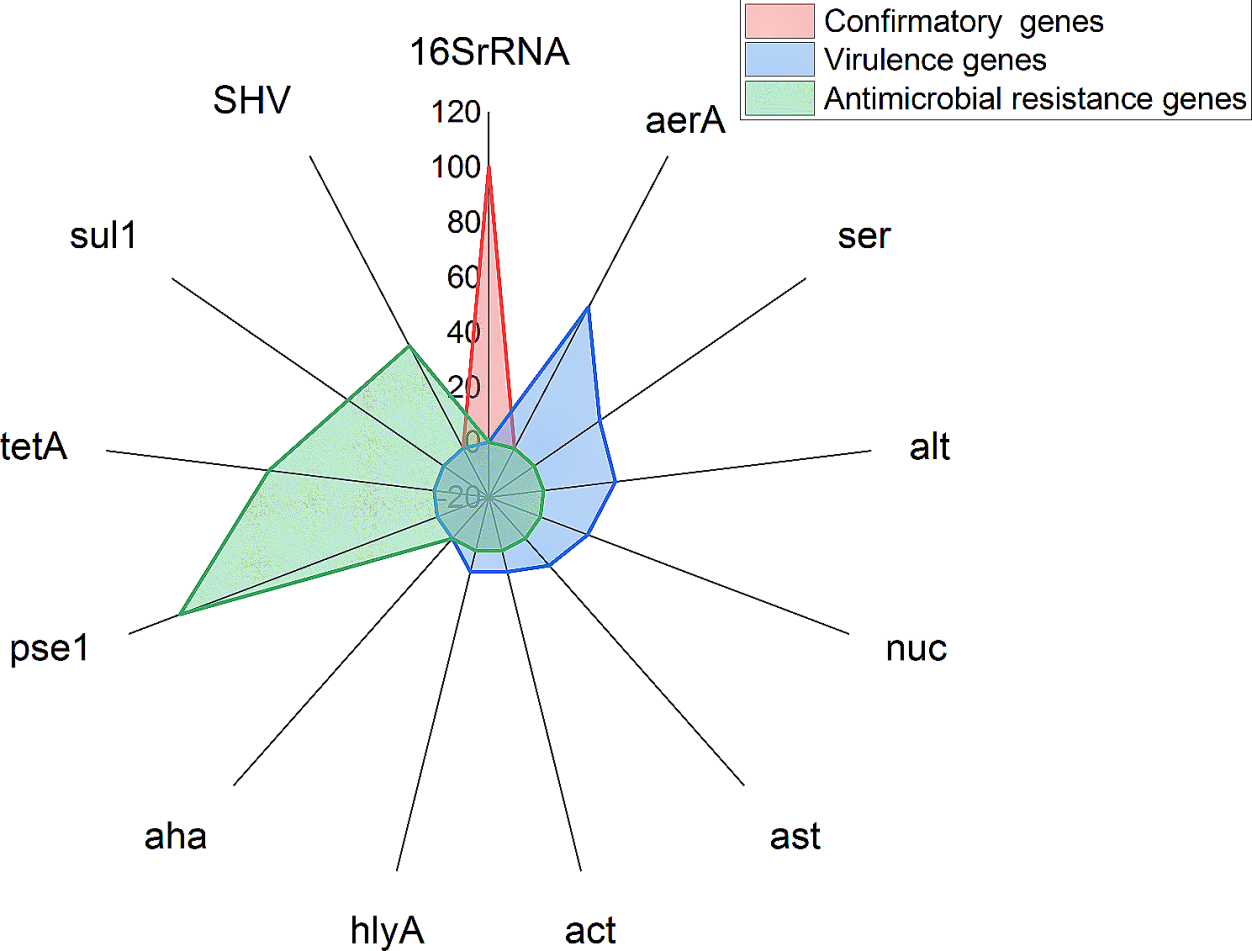
Distribution of different *A.hydrophila* confirmatory, virulence and Antimicrobial resistance genes among recovered isolates

Molecular identification was carried out for all *A. hydrophila* isolates retrieved from diseased fish samples using specific virulence genes primers (*aer*, *act ast*, *alt*, *hly*A, *ser*, *nuc* and *aha*), that produce positive amplicons with a percentage of detected virulence genes in *A. hydrophila* are, *aer*A (22/38, 57.9%), *act* (3/38; 7.9%), *ast* (5/38, 13.1%), *alt* (10/38, 26.3%), *hly*A (3/38, 7.9%), *ser* (11/38, 28.9%) and *nuc* (7/38, 18.4%) respectively. The (*aha*) gene was not detected in any sample as presented in Table 3. and Fig. 5.

**Table 3:** Virulence genes distribution and antimicrobial resistance genes among *A. hydrophila* isolates (*n* = 38)

Gene function	Target gene	Prevalence		Statistical analyses
		No	%	
Confirmatory gene	*16 S rRNA*	38	100	104.73 *P* < 0.0001
Virulence genes	*aerA*	22	57.9	
	*ser*	11	28.9	
	*alt*	10	26.3	
	*ast*	5	13.1	
	*act*	3	7.9	
	*hlyA*	3	7.9	
	*nuc*	7	18.4	
	*aha*	0	0	
Antimicrobial-resistance genes	*β-lactamase pse1*	38	100	15.505 0.001432
	*β-lactamase SHV*	16	42.1	
	*sul*1	16	42.1	
	*tet*A	23	60.5	

Regarding antibiotic resistance, All isolates were positive for *pse*1 (38 /38, 100%), *tet*A (23/38, 60.5%), *sul*1 (16/38, 42.1%) and *bla*_SHV_ (16/38, 42.1%). The tested isolates of *A. hydrophila* revealed a significant difference between antimicrobial resistance genes (*P* < 0.05) and a nonsignificant difference among virulence genes (*P* > 0.05) (Table 3).

### Association between antimicrobial agents, virulence and antimicrobial resistance genes

**Fig. 6:**
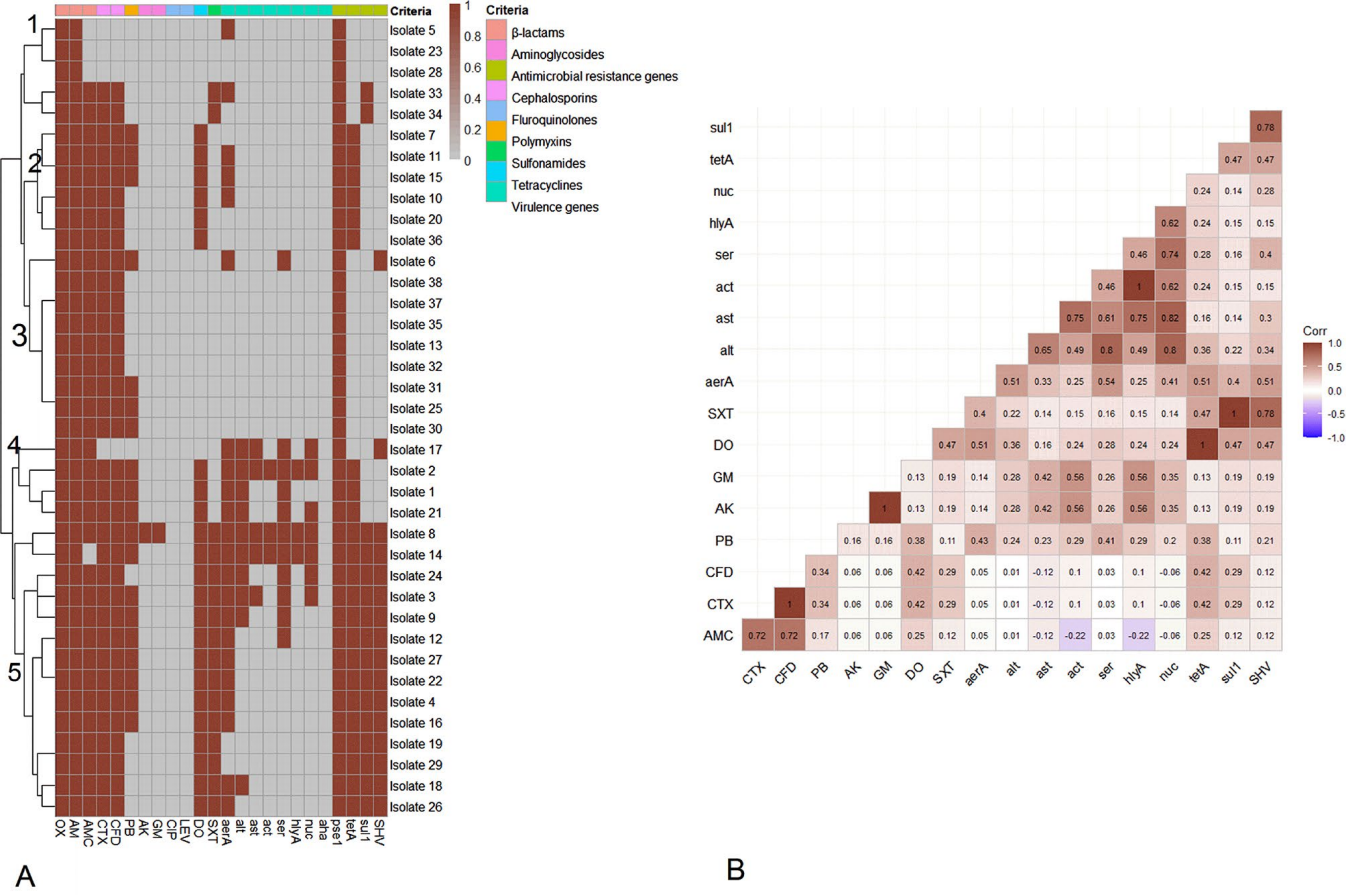
**A** A heatmap of antimicrobial resistance phenotypes, virulence genes, and antimicrobial resistance genes in examined isolates. Dark red squares indicate presence; grey squares indicate absence. The figure shows five clusters (L1–L5). **B** The correlation coefficient (r) between various tested antimicrobial resistance phenotypes, virulence genes, and antimicrobial resistance genes

The relation between the phenotypic multi-drug resistance and the antimicrobial resistance genes for *A. hydrophila* isolates is represented in (Table 4; Fig. 6) and showed a strong correlation between DO and *tet*A (*r* = 1); SXT and *sul*1 (*r* = 1); GM and AK (*r* = 1); CFD and CTX (*r* = 1). The results of the MAR index varied between (0.16–0.83) are shown in Table 4. Heatmap and hierarchical clustering grouped isolates into five clusters (L1, L2, L3, L4, and L5) based on AMR phenotypes, virulence genes, and antimicrobial resistance genes (Fig. 6). L1, L2, and L3 had related isolates, whereas L4 and L5 had other related isolates. Despite no grouping, isolates 25,30 had identical AMR phenotypes, virulence genes, and antimicrobial resistance genes. 28.9% (11/38) of recovered isolates were considered XDR, while 63.1% (24/38) of recovered isolates were considered MDR (Table 4). It was found that some isolates harbor both virulence and MAR genes; where the highest percentage of isolates (5.3%) was carrying 11/12 virulence and resistance genes, followed by (5.3%) isolates harboring 9/12 virulence and resistance genes (Table 5**)**.

**Table 4:** The relation between the phenotypic multi-drug resistance and the antimicrobial resistance genes for *A. hydrophila* isolates

NO. of Isolates	Phenotypic antibiotic resistance	Antimicrobial resistance genes	MAR	
1	OX, AM, AMC, CTX, CFD, PB, DO, SXT, AK, GM	*pse*1, *sul*1, *tet*A, *SHV*	0.83	XDR
1	OX, AM, CTX, CFD, PB, DO, SXT	*pse1, sul1, tetA, SHV*	0.75	XDR
9	OX, AM, AMC, CTX, CFD, PB, DO, SXT	*pse*1, *tet*A, *sul*1, *SHV*	0.66	XDR
6	OX, AM, AMC, CTX, CFD, PB, DO	*pse*1, *tet*A	0.58	MDR
3	OX, AM, CTX, CFD, DO, SXT	*pse*1, *sul*1, *tet*A, *SHV*	0.5	MDR
1	OX, AM, AMC, CTX, CFD, PB	*pse*1, *SHV*	0.5	MDR
2	OX, AM, CTX, CFD, SXT	*pse*1, *sul*1	0.41	MDR
3	OX, AM, CTX, CFD, DO	*pse*1, *tet*A	0.41	MDR
8	OX, AM, CTX, CFD	*pse*1	0.33	MDR
1	OX, AM, AMC	*pse*1, *SHV*	0.25	MDR
3	OX, AM	*pse*1	0.16	DR

**Table 5:** The percentage of isolates harbor both virulence and multiple antibiotic resistance genes

A. hydrophila isolates		Virulence genes (*n* = 8)	Antibiotic-resistant genes Detected (*n* = 4)	Total number of detected genes (*n* = 12)
NO.	%			no
2	5.3	*hly*A, *aer*A, *ser*, *alt, nuc, act*, *ast*,	*pse*1, *tet*A, *sul*1, *bla*_SHV_	11
1	2.6	*hly*A, *aer*A, *ser*, *alt, nuc, act*, *ast*	*pse*1, *tet*A	9
1	2.6	*aer*A, *ser,alt,nuc,ast*	*pse*1, *tet*A, *sul*1, *bla*_SHV_	9
1	2.6	*aer*A, *ser,alt,nuc*	*pse*1, *tet*A, *sul*1, *bla*_SHV_	8
2	5.3	*aer*A, *ser,alt*	*pse*1, *tet*A, *sul*1, *bla*_SHV_	7
1	2.6	*aer*A, *ser,alt,nuc*	*pse*1, *bla*_SHV_	6
1	2.6	*aer*A, *ser,alt,nuc*	*pse*1, *tet*A	6
1	2.6	*aer*A, *alt*	*pse*1, *tet*A, *sul*1, *bla*_SHV_	6
1	2.6	*aer*A, *ser*	*pse*1, *tet*A, *sul*1, *bla*_SHV_	6
5	13.2	*aer*A	*pse*1, *tet*A, *sul*1, *bla*_SHV_	5
1	2.6	*aer*A, *ser,alt*	*pse*1, *tet*A	5
1	2.6	*aer*A, *ser*	*pse*1, *bla*_SHV_	4
2	5.3	*-*	*pse*1, *tet*A, *sul*1, *bla*_SHV_	4
3	7.9	*aer*A	*pse*1, *tet*A	3
1	2.6	*aer*A	*pse*1, *sul*1	3
2	5.3	*-*	*pse*1, *tet*A	2
1	2.6	*-*	*pse*1, *sul*1	2
1	2.6	*aerA*	*pse1*	2
10	26.3	*-*	*pse1*	1

### Pathogenicity test

Seven isolates of *A. hydrophila* were selected for pathogenicity test depending on the prevalence of their virulence genes. The first three experimentally infected groups showed 100% mortality, revealing the high pathogenic capability of the injected strains, Furthermore, the other experimentally infected groups showed mortalities (96.67%, 93.33%, 86.67%, and 80%). The mortality (%) and survivability (%) were calculated as shown in Table (6). The pathogenic strains were re-isolated from freshly dead fish that exhibited high mortalities and the experimentally infected *T. zillii* fish displayed sluggish activity, skin darkening, and dispersed hemorrhagic patches, especially at the base of fins, fin rot, and detached scales. Statistically, there is a significant difference (*P* < 0.5) in the survival rate between different groups.

**Table 6:** Mortality and survivability % in pathogenicity test

Fish group	Virulence genes	Total No. of fish	No of dead fish	Final No of fish	Survival %	Mortality %
**Group 1 Control negative**	-	30	0	30	100%	0
**Group 2** **(Isolate8)**	*hyl*A, *aer*A, *ser*, *alt, nuc, act*, *ast*, *pse*1, *tet*A, *sul*1, *SHV*	30	30	0	0	100%
**Group 3** **(Isolate 14)**	*hyl*A, *aer*A, *ser*, *alt, nuc, act*, *ast*, *pse*1, *tet*A, *sul*1, *SHV*	30	30	0	0	100%
**Group 4** **(Isolate 2)**	*hyl*A, *aer*A, *ser*,*alt,nuc, act*,*ast*,*pse*1,*tet*A	30	30	0	0	100%
**Group 5** **(Isolate 3)**	*aer*A, *ser*,*alt, nuc, ast*, *pse*1, *tet*A, *sul*1, *SHV*	30	29	1	3.33%	96.67%
**Group 6** **(Isolate 24)**	*aer*A, *ser*, *alt, nuc, pse*1, *tet*A, *sul*1, *SHV*	30	28	2	6.67%	93.33%
**Group 7** **(Isolate 17)**	*aer*A, *ser*, *alt, nuc, ast*, *pse*1, *SHV*	30	26	4	13.33%	86.67%
**Group 8 (Isolate 9)**	*aer*A, *ser*, *alt, pse*1, *tet*A, *sul*1, *SHV*	30	24	6	20%	80%

## Discussion

*Aeromonas* species are characterized by their widely ubiquitous distribution in fresh, eustarian, and marine ecosystems, *A. hydrophila* is one of the members of this genus that is most commonly isolated from diseased and apparently healthy fishes [3]. In the present study, moribund fishes that found infected with *A.hydrophila* displayed similar clinical signs and gross lesions as those reported in several previous studies [24, 25]. Also, these results were parallel with the results obtained by Ayoub, et al. [26]; Al-Mokaddem, et al. [1], who found that a clinical examination of the obtained naturally infected Nile tilapia (*Oreochromis niloticus*) with *Aeromonas* species demonstrated abundant hemorrhages, fin and tail fraying, corneal opacity, and body depigmentation. These clinical signs may be attributed to the burst of tiny blood vessels because of the *A. hydrophila* invasion and releasing of the extracellular materials that cause symptoms such as anemia, lethargy, anorexia, ulceration, and hemorrhage. *A. hydrophila* is one of the main pathogens causing Motile *Aeromonas* septicemia in fish and leads to substantial losses in aquaculture [27, 28].

The prevalence of *A. hydrophila* in *T. zillii* and *M. cephalus* was 48.3% and 15% respectively similarly to Jimoh, Jatau [29], Balaji, et al. [30] who reported 47% and 41.7% prevalence percentage of *A. hydrophila* in *Oreochromis niloticus*. The prevalence in internal organs was the highest in liver (42.1%) followed by the kidney (36.8%) and the spleen (13.1%). In contrast, the prevalence in the gills was (7.89%). In the current study, the difference in prevalence percentage could be assigned to fish species, geographical allocation differences, and sampling time [26, 31].

The highest prevalence of *A. hydrophila* among infected *T. zillii* and *M. cephalus* was recorded in the summer season (44.73%), while the lowest was in the autumn (10.52%). This variation may be attributed to the alterations in other water quality parameters in relation to increase in the water temperature, which is considered a stress factor for fish, increasing their susceptibility to infection and aids in bacterial proliferation [32, 33].

Genotypic identification of retrieved *A. hydrophila* isolates using the *16SrRNA* gene is considered an accurate and rapid tool for preliminary bacterial confirmation. In this study, all the isolates carried at least one of the virulent genes. This confirms the high virulence and pathogenicity of *A. hydrophila* isolated from *T. zillii* and *M.cephalus* and their high affinity to cause disease, which matches with the results of the previous researches [34, 35]. In regards to the detected virulent genes, Aerolysin *(aer*A*)* gene was the most frequently detected virulence gene in isolated *A. hydrophila* strains, this comes in agreement with other studies [16, 24, 34]. Aerolysin plays an important role in the pathogenesis of *A. hydrophila* as a pore-forming toxin that destroys membrane permeability, causing osmotic lysis that ends with cell death [36]. Motile Aeromonads, potential foodborne pathogens, require aerolysin (*aer*) and cytotoxic enterotoxins as *act*, *alt* and *ast* genes as *Aeromonas* heat-labile and heat-stable cytotonic enterotoxins. Type II secreted pore-forming cytotoxic enterotoxin gene (*act*) encodes cytotoxic and cytolytic proteins [37]. Five isolates of *A. hydrophila* with a prevalence of 13.1% harbor the *ast* gene, which increases intestinal vascular permeability and intestinal mucosal detachment, in contrast to Ramadan, et al. [16], who identified the *ast* gene in 46% of *Mugil cephalus* isolates, and El-Bahar, et al. [24], who couldn’t detect the *ast* gene in any sample of the Aeromonas isolates from Nile tilapia.

The *alt* gene was detected in ten out of thirty eight *A.hydrophila* isolates, which was lower than those reported by Rather, et al. [38]. Heat-labile enterotoxin (*alt*) induces intestinal fluid retention in animals [39]. Our study revealed that the *hly*A gene was detected in only three strains of *A. hydrophila* with a prevalence of 7.9%, this result is lower than those reported by Hayati, et al. [40]; Simon, et al. [41] who confirmed the presence of *hyl*A in 95% and 39% of *A. hydrophila* isolates, respectively. Protease activity is crucial to *Aeromonas* spp. pathogenesis as it causes tissue damages, or activates toxins and overcomes host defenses [42]. Regarding to serine protease (*ser*) gene, nine isolates were detected with a total prevalence of 28.9% and this result is lower than those found by Yu, Chu [43] and Abu-Elala, et al. [44] who reported a higher percent of the *ser* gene (89%) and (55%) respectively detected in *Aeromonas* isolates. More frequently than in environmental samples, the nuclease (*nuc*) gene has been determined to be a virulence factor in clinical samples [45]. . The total prevalence of *nuc* gene among isolated *Aeromonas* spp was 18.4% lower than those described by Onuk, et al. [46] who detected the *nuc* gene in 54.54% of *Aeromonas* isolates.

The Bacteria frequently possess virulence and antimicrobial-resistance genes on the chromosome or on mobile genetic elements such as plasmids, transposons, and integrons [47]. This association is significant because these genes’ successive acquisition and expression may affect bacterial fitness and host survival [48, 49]. Janda, Abbott [50] noted that bacteria can express up to three β-lactamases through a coordinated process. β-lactam antibiotics cure bacterial illnesses best. However, resistant bacterial strains produce β-lactamases, reducing their efficacy. *Aeromonas* has a β-lactamase gene, which hydrolyzes the β-lactam ring to inactivate the antibiotic [51]. As shown in Table (2), 100% and 42.1% of *A. hydrophila* isolates possessed *bla*_pse1_ and *bla*_SHV_ genes, respectively, which confirmed that genes that code for β-lactamase increase resistance to β-lactam antimicrobials (penicillins and derivatives, cephalosporins, carbapenems, and monobactams), SHV enzymes can hydrolyze monobactams and carbapenems due to modifications in amino acids that alter the active site structure of β-lactamases [50, 52].

The present study also revealed the presence of genes encoding resistance to tetracyclines (*tetA*) and sulfonamides (*sul 1*) in 60.5% and 42.1% of *A. hydrophila* isolates, respectively. The resistance gene of *sul1* had been detected at a high rate of 87.1% and 75% respectively in *Aeromonas* spp. isolated from rainbow trout, *Oreochromis niloticus* and *Clarias gariepinus* [53, 54]. In another study, although *sul1* was present in *A. hydrophila* (41%, 7/17), it could not be detected in *A. sobria* and *A. caviae* [55]. While variable occurrences of the *tet*A (A) resistance gene had been reported in *Aeromonas* from several studies 50%, 87.5% [53], and 67.44% [56].

Antibiotic susceptibility assessments are critical to monitoring the severity of antibiotic resistance and choosing the appropriate drugs for disease treatments in aquaculture to minimize risks to human health. In intensive aquaculture systems, antimicrobial agents are extensively used to control infectious diseases and are often unregulated [57]. All the tested isolates in this study were sensitive (100%) to levofloxacin and ciprofloxacin. In contrast, lower resistance (11.2%) to amikacin and gentamicin agrees with the results obtained by Ramadan, et al. [16] who demonstrated lower resistance to gentamicin for the bacterial isolates from fish samples. *Aeromonas* species are susceptible to Fluoroquinolones [50]. Ciprofloxacin is known to be the most effective treatment for most diseases. The high levels of resistance to ampicillin and oxacillin were identical to those reported by [58]. They observed that all the isolates tested were highly resistant to amoxicillin and ampicillin. Similar results have been reported in isolates borne on zebrafish and Nile tilapia [59].

Our results revealed a high prevalence of MAR in *A. hydrophila* isolates from freshwater and saltwater fish in Egypt. The higher frequencies of antibiotic resistance of the isolates may imply that antimicrobial agents are used more frequently in aquaculture. The multiple antibiotic resistance index (MAR) has been used to specify the degree of antibiotic use. The value of the MAR index is higher than 0.2 reflecting the bacterial isolates from high-risk sources of antibiotic contagion where antibiotics are frequently used. Higher values of the MAR index (> 0.2 to 0.93) were expressed by Krumperman [22], Tartor, et al. [35], Vivekanandhan, et al. [60], who noted that MAR indices were displayed in 87.2% of *A. hydrophila* isolates. These results nearly agree with those captured by Kusdarwati, et al. [61]. Depending on the antibiotic resistance phenotype, 63.16% (24\38) of tested *A. hydrophila* isolates exhibited multidrug resistance (MDR) to five or more antimicrobial classes and 28.95% (11/38) of tested isolates exhibited extensive drug resistance (XDR) to eleven or more antimicrobial classes, these results agreed with those found by Algammal, et al. [62]; Algammal, et al. [12]. Isolates that demonstrated resist at least one agent in all antibiotics from multiple classes (except for 1 or 2) are categorized as XDR. Isolates that demonstrated resistance to three or more drugs were categorized as MDR, as previously documented. [33, 75].

## Conclusion

Our findings showed that most recovered *A. hydrophila* isolates from the Suez Canal area, Egypt carried both virulence and antibiotic-resistant genes. It showed that the prevalence and distribution of various virulence and antibiotic-resistant genes in *A. hydrophila* is crucial in the occurrence of the septicemic disease, furthermore, the presence of such antibiotic-resistant strains in aquaculture will be a constrain in treatment or even control of infected fishes. In addition, these findings raise a public health concern regarding the illegal use of antibiotics in fish farms and the expected human health implications.

## Data Availability

Data and materials are available upon request.

## Declarations

All protocols including live animals were approved by the Animal Ethics Review Committee of Suez Canal University guidelines (AERC-SCU 2023026).

## References

[1] Al-Mokaddem AK, Abdel-moneam DA, Ibrahim RA, Saleh M, Shaalan M (2022). Molecular identification, histopathological analysis and immunohistochemical characterization of non-pigmented *Aeromonas salmonicida* subsp. Salmonicida in *Mugil Carinatus* (Valenciennes, 1836). Aquacult Rep.

[2] Sreedharan K, Philip R, Singh ISB (2012). Virulence potential and antibiotic susceptibility pattern of motile aeromonads associated with freshwater ornamental fish culture systems: a possible threat to public health. Braz J Microbiol.

[3] Dias MK, Sampaio LS, Proietti-Junior AA, Yoshioka ET, Rodrigues DP, Rodriguez AF (2016). Lethal dose and clinical signs of *Aeromonas hydrophila* in *Arapaima gigas* (Arapaimidae), the giant fish from Amazon. Vet Microbiol.

[4] Hu M, Wang N, Pan Z, Lu C, Liu Y (2012). Identity and virulence properties of *Aeromonas isolates* from diseased fish, healthy controls and water environment in China. Lett Appl Microbiol.

[5] Zhang D, Moreira GS, Shoemaker C, Newton JC, Xu D-H (2016). Detection and quantification of virulent *Aeromonas hydrophila* in channel catfish tissues following waterborne challenge. FEMS Microbiol Lett.

[6] Beaz-Hidalgo R, Figueras M (2013). *Aeromonas Spp* whole genomes and virulence factors implicated in fish disease. J Fish Dis.

[7] Chacón M, Figueras M, Castro-Escarpulli G, Soler L, Guarro J (2003). Distribution of virulence genes in clinical and environmental isolates of *Aeromonas spp*. Antonie Van Leeuwenhoek.

[8] Puthucheary S, Puah SM, Chua KH (2012). Molecular characterization of clinical isolates of *Aeromonas species* from Malaysia. PLoS ONE.

[9] Yousr A, Napis S, Rusul G, Son R (2007). Detection of aerolysin and hemolysin genes in *Aeromonas spp* isolated from environmental and shellfish sources by polymerase chain reaction. ASEAN Food J.

[10] Pridgeon JW, Klesius PH (2011). Molecular identification and virulence of three *Aeromonas hydrophila* isolates cultured from infected channel catfish during a disease outbreak in west Alabama (USA) in 2009. Dis Aquat Org.

[11] Watts JE, Schreier HJ, Lanska L, Hale MS (2017). The rising tide of antimicrobial resistance in aquaculture: sources, sinks and solutions. Mar Drugs.

[12] Algammal AM, Mabrok M, Ezzat M, Alfifi KJ, Esawy AM, Elmasry N (2022). Prevalence, antimicrobial resistance (AMR) pattern, virulence determinant and AMR genes of emerging multi-drug resistant *Edwardsiella tarda* in Nile tilapia and African catfish. Aquaculture.

[13] Rhodes G, Huys G, Swings J, Mcgann P, Hiney M, Smith P (2000). Distribution of oxytetracycline resistance plasmids between aeromonads in hospital and aquaculture environments: implication of Tn 1721 in dissemination of the tetracycline resistance determinant Tet A. Appl Environ Microbiol.

[14] Chen L, Zhang H, Liu Q, Pang X, Zhao X, Yang H (2019). Sanitising efficacy of lactic acid combined with low-concentration sodium hypochlorite on Listeria innocua in organic broccoli sprouts. Int J Food Microbiol.

[15] Yano Y, Hamano K, Tsutsui I, Aue-Umneoy D, Ban M, Satomi M (2015). Occurrence, molecular characterization, and antimicrobial susceptibility of *Aeromonas spp* in marine species of shrimps cultured at inland low salinity ponds. Food Microbiol.

[16] Ramadan H, Ibrahim N, Samir M, Abd El-Moaty A, Gad T (2018). *Aeromonas hydrophila* from marketed mullet (*Mugil cephalus*) in Egypt: PCR characterization of β‐lactam resistance and virulence genes. J Appl Microbiol.

[17] Austin B, Austin DA (2012). Bacterial fish pathogens, Disease of Farmed and Wild Fish.

[18] Quinn PJ, Markey BK, Carter ME, Donnelly WJC, Leonard FC (2002). Veterinary microbiology and microbial disease.

[19] Arai T, Komatsu S, Komatsu Y (1981). Extracellular protease production of various bacteria and the role of proteases on the pathogenicity of opportunistic pathogens. Keio J Med.

[20] Kumar S, Stecher G, Li M, Knyaz C, Tamura K (2018). MEGA X: molecular evolutionary genetics analysis across computing platforms. Mol Biol Evol.

[21] Smith P, Egan S (2018). Standard protocols for antimicrobial susceptibility testing of Vibrionaceae isolated from aquatic animals. Bull Eur Assoc Fish Pathol.

[22] Krumperman PH (1983). Multiple antibiotic resistance indexing of *Escherichia coli* to identify high-risk sources of fecal contamination of foods. Appl Environ Microbiol.

[23] Marzouk M, Moustafa M, Mohamed NM. Evaluation of immunomodulatory effects of some probiotics on cultured *Oreochromis niloticus*. In: *8th International symposium on tilapia in aquaculture: 2008*; 2008.

[24] El-Bahar HM, Ali NG, Aboyadak IM, Khalil SAES, Ibrahim MS (2019). Virulence genes contributing to *Aeromonas hydrophila* pathogenicity in *Oreochromis niloticus*. Int Microbiol.

[25] Omar A, Moustafa EM, Zayed MM (2016). Identification and characterization of virulence associated genes from pathogenic *Aeromonas hydrophila* strains. World Vet J.

[26] Ayoub HF (2021). Isolation, identification and antimicrobial profile of *Aeromonas spp*, *Pseudomonas spp* and *Vibrio Spp* from the Nile Tilapia, *Oreochromis niloticus* in fish farms. Egypt J Aquat Biol Fish.

[27] Moustafa M, Mohamed L, Mahmoud M, Soliman W, El-Gendy M (2010). Bacterial infections affecting marine fishes in Egypt. J Am Sci.

[28] Aoki T (2011). Fish diseases and disorders, volume 3: viral, bacterial and fungal infections - CABI.org. Wallingford.

[29] Jimoh S, Jatau E (2010). Prevalence of *Aeromonas hydrophila* in tilapia obtained from Ahmadu Bello University, Zaria dam, Nigeria. Sci Focus.

[30] Balaji V, Jesudason MV, Sridharan G (2004). Cytotoxin testing of environmental *Aeromonas spp* in Vero cell culture. Indian J Med Res.

[31] Pretto-Giordano LG, Müller EE, Freitas, JCd (2010). Silva VGd. Evaluation on the Pathogenesis of Streptococcus agalactiae in Nile Tilapia (*Oreochromis niloticus*). Braz Arch Biol Technol.

[32] Hussain R (2002). Studies on some bacterial infections affecting certain marine fishes in the Arabian Gulf of Kingodom of Saudi Arabia. Bull Japanese Soc Sci Fishers.

[33] Zorrilla I, Chabrillón M, Arijo S, Dıaz-Rosales P, Martınez-Manzanares E, Balebona M (2003). Bacteria recovered from diseased cultured gilthead sea bream (*Sparus aurata* L.) in southwestern Spain. Aquaculture.

[34] Oliveira ST, Veneroni-Gouveia G, Costa MM (2012). Molecular characterization of virulence factors in *Aeromonas hydrophila* obtained from fish. Pesquisa Veterinária Brasileira.

[35] Tartor YH, EL-Naenaeey E-SY, Abdallah HM, Samir M, Yassen MM, Abdelwahab AM (2021). Virulotyping and genetic diversity of *Aeromonas hydrophila* isolated from Nile tilapia (*Oreochromis niloticus*) in aquaculture farms in Egypt. Aquaculture.

[36] Iacovache I, De Carlo S, Cirauqui N, Dal Peraro M, van der Goot FG, Zuber B (2016). Cryo-EM structure of aerolysin variants reveals a novel protein fold and the pore-formation process. Nat Commun.

[37] Sha J, Kozlova E, Chopra A (2002). Role of various enterotoxins in *Aeromonas hydrophila*-induced gastroenteritis: generation of enterotoxin gene-deficient mutants and evaluation of their enterotoxic activity. Infect Immun.

[38] Rather M, Willayat M, Wani S, Hussain S, Shah S (2019). Enterotoxin gene profile and molecular epidemiology of *Aeromonas species* from fish and diverse water sources. J Appl Microbiol.

[39] Chopra AK, Houston CW (1999). Enterotoxins in Aeromonas-associated gastroenteritis. Microb Infect.

[40] Hayati HR, Hassan M, Ong B, Abdelhadi Y, Hidayahanum HN, Sharifah R (2015). Virulence genes detection of *Aeromonas hydrophila* originated from diseased freshwater fishes. Adv Environ Biology.

[41] Simon SS, Lalitha K, Joseph TC (2016). Virulence properties of *Aeromonas spp* from modified-atmosphere-and vacuum-packed milk fish (*Chanos chanos* Forsskal, 1775). Ann Microbiol.

[42] Tomás J. The main Aeromonas pathogenic factors. International Scholarly Research Notices. 2012; 2012:256261.

[43] Yu C-P, Chu K-H (2011). Molecular quantification of virulence gene-containing Aeromonas in water samples collected from different drinking water treatment processes. Environ Monit Assess.

[44] Abu-Elala NM, Samir A, Wasfy M, Elsayed M (2019). Efficacy of injectable and immersion polyvalent vaccine against streptococcal infections in broodstock and offspring of Nile tilapia (*Oreochromis niloticus*). Fish Shellfish Immunol.

[45] Dodd HN, Pemberton JM (1996). Cloning, sequencing, and characterization of the nucH gene encoding an extracellular nuclease from *Aeromonas hydrophila* JMP636. J Bacteriol.

[46] Onuk EE, Findik A, Turk N, Altun S, Korun J, Ozer S (2013). Molecular identification and determination of some virulence genes of Aeromonas spp. in fish and water from Turkish coastal regions. Revue Médecine Véterinaire.

[47] Kottara A, Hall JP, Harrison E, Brockhurst MA (2018). Variable plasmid fitness effects and mobile genetic element dynamics across Pseudomonas species. FEMS Microbiol Ecol.

[48] da Silva GJ, Mendonça N (2012). Association between antimicrobial resistance and virulence in *Escherichia coli*. Virulence.

[49] Beceiro A, Tomás M, Bou G (2013). Antimicrobial resistance and virulence: a successful or deleterious association in the bacterial world?. Clin Microbiol Rev.

[50] Janda JM, Abbott SL (2010). The genus Aeromonas: taxonomy, pathogenicity, and infection. Clin Microbiol Rev.

[51] Bradford PA (2001). Extended-spectrum βlactamases in the 21st century: characterization, epidemiology, and detection of this important resistance threat. Clin Microbiol Rev.

[52] Chen P-L, Lamy B, Ko W-C (2016). *Aeromonas dhakensis*, an increasingly recognized human pathogen. Front Microbiol.

[53] Ndi O, Barton M (2011). Incidence of class 1 integron and other antibiotic resistance determinants in *Aeromonas spp* from rainbow trout farms in Australia. J Fish Dis.

[54] Ashraf A, Ahmed A, Fatma I, Amany O, Emad E (2017). Molecular studies on antibiotic resistant genes of Aeromonas species isolated from fish. Nat Sci.

[55] Okolie CA (2015). Characterization of antimicrobial resistance genes of *Aeromonas spp* isolated from fish and investigation of phytochemical treatment efficacy against resistant isolates.

[56] Hossain S, Dahanayake PS, De Silva BC, Wickramanayake M, Wimalasena S, Heo GJ (2019). Multidrug resistant *Aeromonas spp* isolated from zebrafish (*Danio rerio*): Antibiogram, antimicrobial resistance genes. Lett Appl Microbiol.

[57] Heuer OE, Kruse H, Grave K, Collignon P, Karunasagar I, Angulo FJ (2009). Human health consequences of use of antimicrobial agents in aquaculture. Clin Infect Dis.

[58] Tayler AE, Ayala JA, Niumsup P, Westphal K, Baker JA, Zhang L (2010). Induction of β-lactamase production in *Aeromonas hydrophila* is responsive to β-lactam-mediated changes in peptidoglycan composition. Microbiology.

[59] Hossain S, De Silva B, Dahanayake P, Heo GJ (2018). Characterization of virulence properties and multi-drug resistance profiles in motile *Aeromonas spp* isolated from zebrafish (*Danio rerio*). Lett Appl Microbiol.

[60] Vivekanandhan G, Savithamani K, Hatha A, Lakshmanaperumalsamy P (2002). Antibiotic resistance of *Aeromonas hydrophila* isolated from marketed fish and prawn of South India. Int J Food Microbiol.

[61] Kusdarwati R, Dinda N, Nurjanah I. Antimicrobial resistance prevalence of *Aeromonas hydrophila* isolates from motile *Aeromonas septicemia* disease. In: *IOP Conference Series: Earth and Environmental Science*: 2018: IOP Publishing; 2018: 012076.

[62] Algammal AM, Mabrok M, Sivaramasamy E, Youssef FM, Atwa MH, El-Kholy AW (2020). Emerging MDR-*Pseudomonas aeruginosa* in fish commonly harbor oprL and toxA virulence genes and blaTEM, blaCTX-M, and tetA antibiotic-resistance genes. Sci Rep.

[63] Stackebrandt E, Murray R, Trüper H (1988). Proteobacteria Classis nov., a name for the phylogenetic taxon that includes the purple bacteria and their relatives. Int J Syst Evol Microbiol.

[64] Singh V, Rathore G, Kapoor D, Mishra B, Lakra W (2008). Detection of aerolysin gene in *Aeromonas hydrophila* isolated from fish and pond water. Indian J Microbiol.

[65] Nam I-Y, Joh K (2007). Rapid detection of virulence factors of Aeromonas isolated from a trout farm by hexaplex-PCR. J Microbiol.

[66] Rahayu K, Daruti D. Detection and analysis of hemolysin genes in *Aeromonas hydrophila* isolated from Gouramy (*Osphronemus gouramy*) by polymerase chain reaction (PCR). In: *IOP Conference Series: Earth and Environmental Science*: 2018: IOP Publishing; 2018: 012001.

[67] Li T, Raza S, Yang B, Sun Y, Wang G, Sun W (2020). Aeromonas Veronii Infection in Commercial Freshwater Fish: a potential threat to Public Health. Animals.

[68] Igbinosa IH, Okoh AI. Antibiotic susceptibility profile of *Aeromonas species* isolated from wastewater treatment plant. The scientific world journal. 2012; 2012:764563. 10.1100/2012/764563 PMC3425809 22927788

[69] Colom K, Pérez J, Alonso R, Fernández-Aranguiz A, Lariño E, Cisterna R (2003). Simple and reliable multiplex PCR assay for detection of bla TEM, Bla SHV and Bla OXA–1 genes in *Enterobacteriaceae*. FEMS Microbiol Lett.

[70] Pei R, Kim S-C, Carlson KH, Pruden A (2006). Effect of river landscape on the sediment concentrations of antibiotics and corresponding antibiotic resistance genes (ARG). Water Res.

[71] Wu N, Qiao M, Zhang B, Cheng W-D, Zhu Y-G (2010). Abundance and diversity of tetracycline resistance genes in soils adjacent to representative swine feedlots in China. Environ Sci Technol.

